# Latent inflammatory activity is associated with alterations in iron bioavailability during early postpartum adaptation in dairy cows

**DOI:** 10.3389/fvets.2026.1923985

**Published:** 2026-09-15

**Authors:** Marko Cincović, Nikolina Milošević, Mira Majkić, Jaka Jakob Hodnik, Branka Kramberger, Jože Starič

**Affiliations:** 1 Laboratory of Pathophysiology, Department of Veterinary Medicine, Faculty of Agriculture, University of Novi Sad, Novi Sad, Serbia; 2 Veterinary Faculty, University of Ljubljana, Ljubljana, Slovenia

**Keywords:** acute phase proteins, blood, cytokine, Fe, inflammation, principal component analysis

## Abstract

**Introduction:**

The transition from late gestation to early lactation is accompanied by profound metabolic and immunological adaptations in dairy cows. While the relationship between inflammation and iron metabolism has been extensively studied in clinical diseases, less is known about how physiological or low-grade inflammatory processes influence iron bioavailability during early lactation. This study investigated associations between inflammatory activity and indicators of iron bioavailability in dairy cows during the early postpartum period, with particular emphasis on the use of a latent inflammatory index derived by principal component analysis (PCA).

**Methods:**

Twenty clinically healthy dairy cows were monitored on Days 0, 3, 7, 14, and 21 postpartum. Blood serum concentrations of inflammatory markers (IL-1, IL-6, TNF-*α*, IFN-*γ*, haptoglobin, serum amyloid A, and extracellular HSP70) and iron-related parameters (Fe, TIBC, UIBC, and transferrin saturation, TS%) were determined. Temporal changes were evaluated using multivariate analyses, correlation analysis, PCA, and linear mixed-effects models.

**Results:**

Iron-related parameters declined during early lactation, reaching minimum values around Day 7, followed by partial recovery by Day 21. In contrast, inflammatory markers exhibited a transient increase, peaking between Days 3 and 7 before gradually returning toward baseline. Multivariate clustering and heatmap analyses revealed a clear temporal separation between the inflammatory activation phase and the recovery phase, indicating an inverse relationship between inflammatory status and iron bioavailability. Correlation analysis confirmed significant negative associations between inflammatory mediators, particularly IL-6 and haptoglobin, and all iron metabolism indicators. Because of substantial multicollinearity among inflammatory variables, PCA was applied to generate a latent inflammatory score. The first principal component (PC1-infla) explained 63.5% of the total variance and represented overall systemic inflammatory activity. Linear mixed-effects models demonstrated that higher PC1-infla scores were significantly associated with lower Fe, TIBC, UIBC, and TS% values (*p* < 0.001).

**Discussion:**

PC1-infla was consistently associated with markers of iron bioavailability and showed the strongest statistical association with iron-related biomarkers among the evaluated inflammatory variables. These findings indicate that early postpartum adaptation in dairy cows is characterized by coordinated inflammatory activation accompanied by reduced iron bioavailability. The overall inflammatory burden appears to play a greater role in regulating iron metabolism than individual inflammatory markers alone.

## 1 Introduction

Biomarkers are objectively measurable biological characteristics that reflect physiological, pathological, or pharmacological processes and are widely used to assess animal health and adaptation. Among these, inflammatory biomarkers have gained increasing attention for monitoring the transition period in dairy cows. Inflammation during the periparturient period is characterized by activation of a cytokine cascade, induction of the acute-phase response, and alterations in extracellular heat shock protein concentrations, with the magnitude of these changes depending on the health status of the animals (1, 2). The TNF-*α*/IL-1/IL-6/acute-phase protein cascade and the synergistic interactions among its components are well established (3–6).

The periparturient period in dairy cows is characterized by profound physiological adaptations associated with parturition and the onset of lactation, resulting in substantial metabolic stress and the establishment of a negative energy balance (7). During this period, nutrients are preferentially redirected toward milk synthesis, accompanied by increased adipose tissue mobilization, elevated concentrations of non-esterified fatty acids (NEFA) and ketone bodies, and increased hepatic and oxidative burden. Collectively, these changes constitute the basis for the development of metabolic low-grade inflammation (8, 9). Gestation, parturition, lactation, and metabolically induced inflammation during the transition period are closely interconnected, as they influence major endocrine pathways, including the ACTH/cortisol and growth hormone/IGF-1 axes, modulate tissue macrophage activity, especially in adipose tissue and affect cellular integrity and redistribution of nutrients (10, 11).

Tumor necrosis factor-alpha (TNF-*α*) and interleukin-1 (IL-1) are among the earliest mediators released following macrophage activation and play a central role in initiating the inflammatory response. TNF-*α* promotes the expression of adhesion molecules, leukocyte migration, and the production of additional cytokines, particularly IL-6, whereas IL-1 acts synergistically with TNF-*α* to amplify inflammatory signaling. Interleukin-6 (IL-6) is a key mediator of the acute-phase response and stimulates hepatocytes to synthesize acute-phase proteins. Among these proteins, serum amyloid A (SAA) and haptoglobin (Hp) are considered the most important biomarkers in cattle (12).

Serum amyloid A is a highly sensitive and early marker of inflammation, with concentrations increasing several-fold within hours of inflammatory activation. It contributes to immune cell recruitment, modulation of inflammatory processes, and lipid transport during the acute phase. Haptoglobin is synthesized more gradually than SAA; however, its concentrations remain elevated for a longer period. Its primary function is to bind free hemoglobin and prevent oxidative damage, while also serving as a valuable indicator of systemic inflammation in cattle. Additional mediators also play important roles in inflammatory response. Interferon-gamma (IFN-*γ*), a cytokine primarily produced by T lymphocytes and natural killer (NK) cells, is closely associated with cellular immunity and enhances cytokine production through macrophage activation (13). In addition, once released into the extracellular environment, heat shock protein 70 (Hsp70) acts as a damage associated molecular pattern (DAMP) molecule that alerts the immune system to cellular stress or damage. Through its chaperokine activity, extracellular Hsp70 modulates cytokine production and promotes immune cell activation, thereby initiating sterile inflammatory responses and enhancing pre-existing inflammation (14).

Iron concentration decreases significantly during inflammatory responses in dairy cows (15, 16). There are several significant mechanisms linking inflammation and iron depletion (17, 18). Activation of the immune system leads to the release of pro-inflammatory cytokines, especially IL-6, which stimulates the synthesis of hepcidin in the liver. Hepcidin is the principal regulator of iron homeostasis and acts by inhibiting ferroportin, thereby preventing the export of iron from enterocytes and macrophages into the circulation. Consequently, iron is retained within cells of the reticuloendothelial system, resulting in decreased serum iron concentrations. This mechanism represents an adaptive host defense strategy that restricts iron availability to invading microorganisms. In addition, TNF-*α* and IL-1 suppress the synthesis of transferrin, a negative acute-phase protein, thereby further influencing iron metabolism.

Iron bioavailability and status are commonly evaluated through the determination of serum iron (Fe), total iron-binding capacity (TIBC), unsaturated iron-binding capacity (UIBC), and transferrin saturation percentage (TS%) (19). These parameters provide complementary information regarding circulating iron concentrations, iron transport capacity, transferrin saturation, and reserve iron-binding capacity. Their interpretation is essential for distinguishing between inflammation-induced hypoferremia and true iron deficiency (19, 20). Serum iron reflects the concentration of circulating iron bound to transferrin, and decreased serum iron levels indicate reduced iron availability resulting from inflammation, nutritional deficiency, or impaired mobilization from body stores. TIBC represents the overall capacity of transferrin to bind iron and indirectly reflects transferrin concentration. Elevated TIBC values are characteristic of iron deficiency, whereas during inflammation they are often reduced due to decreased transferrin synthesis. Transferrin saturation (TS%) is calculated as the ratio of serum iron to TIBC and reflects the degree to which the iron transport capacity of transferrin is utilized. Low TS% values indicate reduced iron availability to tissues and may occur both during inflammation, owing to decreased serum iron concentrations, and in iron deficiency, where elevated TIBC further contributes to lower transferrin saturation.

Alterations in iron bioavailability accompany the inflammatory response during early lactation (21). However, previous studies investigating the relationship between inflammation and iron metabolism have primarily focused on overt clinical diseases (16, 22, 23), whereas subclinical inflammatory processes and their metabolic consequences remain insufficiently explored. Previous investigations were based on determining the links between individual inflammatory parameters with parameters of iron metabolism (24). To date, there is a lack of data regarding the use of complex models or latent indices that would explain joint inflammatory action during physiological or low grade inflammation.

The development of such indices is physiologically justified by the fact that pro-inflammatory cytokines, acute-phase proteins, and extracellular chaperones exhibit coordinated changes and act synergistically during inflammation in early lactation (6). Furthermore, these indices have a strong statistical rationale, as conventional statistical approaches often struggle to disentangle the shared and individual contributions of variables that are highly correlated and exhibit substantial multicollinearity, potentially leading to the loss of valuable information (25).

Several indices have previously been used to assess inflammatory status in dairy cows, including the neutrophil-to-lymphocyte ratio (NLR) and platelet-to-lymphocyte ratio (PLR) (26), the liver functionality index (27), and integrative multivariate approaches combining multiple biomarkers for the assessment of mastitis and laminitis (28). In human medicine, the use of PCA in the assessment of the “cytokine fingerprint” has been shown to have great diagnostic value, and numerous models have been developed for the assessment of cytokine storm syndromes, particularly in patients with COVID-19 (29). Although several inflammatory indices have been proposed in veterinary medicine, most are based on predefined combinations of biomarkers or hematological ratios. In contrast, the present approach derives a latent inflammatory index directly from the covariance structure of multiple inflammatory biomarkers. By integrating the common biological signal while reducing multicollinearity, this PCA-derived score provides a statistically robust representation of the overall inflammatory burden rather than relying on a single biomarker or arbitrarily weighted index.

Therefore, the aim of the present study was to investigate the relationships between inflammatory parameters (IL-1, IL-6, TNF-*α*, IFN-*γ*, Hp, SAA, and eHSP70) and indicators of iron bioavailability (Fe, TIBC, UIBC, and TS%) in cows in early lactation, using different models. Particular emphasis was placed on evaluating the utility of a PCA-derived latent inflammatory score as an indicator of overall inflammatory burden and its association with iron bioavailability parameters during early lactation.

## 2 Materials and methods

### 2.1 Experimental animals and study design

The experiment was conducted in Vojvodina Province, northern Serbia, during April and early May, a thermoneutral period. A total of 20 clinically healthy Holstein-Friesian cows at the beginning of second or third lactation were included in the research. The cows that were in optimal body condition (BCS 3.25–3.5) and showed no signs of dystocia or any health disorders throughout the experimental period (clinically examined by a veterinarian) were included in the study. Cows that did not meet the predefined inclusion criteria, including the required body condition score, were excluded before the final study population was established. Consequently, the study included 20 cows that fulfilled all selection criteria. The study was conducted on a large dairy farm with a free-stall system and cubicles, housing 2,000 animals. The average milk yield per cow on the farm was 8,500 kg per standard lactation. Throughout the study, the experimental dairy cows were housed under standard farm conditions and were provided with a total mixed ration (TMR) twice daily, with unrestricted access to drinking water. The diet was specifically formulated to meet the nutritional requirements of cows (30) during early lactation and supplied approximately 21.5 kg of dry matter (DM), 153.2 MJ of net energy for lactation, and 18.3% crude protein on a DM basis. The ration also contained 39.7% rumen undegradable protein relative to crude protein, 4.9% fat, 17.2% crude fiber, 22.6% acid detergent fiber (ADF), and 37.2% neutral detergent fiber (NDF). Overall, the feeding program was designed to achieve an average intake of approximately 23 kg DM per day, corresponding to about 43 kg of fresh feed. No additional dietary fat or energy supplementation beyond the standard transition diet was provided during late gestation.

The study was designed as a longitudinal experiment with repeated measurements, in order to enable the monitoring of dynamic changes of biomarkers in the critical postpartum period. Sampling was performed at five time points: Day 0 (calving), Day 3, Day 7, Day 14 and Day 21 after calving. Each cow represented an independent experimental unit, while time points were considered as repeated measurements within individuals.

### 2.2 Biological material and laboratory analyses

Blood was sampled from the coccygeal vein, immediately after the morning milking. Vacutainers with clot activator for serum separation Becton, Dickinson and Company (BD), USA were used for blood collection. The tubes were placed in a portable refrigerator, and the spontaneously separated serum was analyzed for blood parameters on the same day after sampling.

The following parameters of iron metabolism were analyzed: serum iron (Fe), total iron binding capacity (TIBC), unsaturated iron binding capacity (UIBC) and transferrin saturation (TS%). Biochemical analyses were performed with a Chemray analyzer automatic biochemical analyzer (Rayto, People’s Republic of China) using standard kits (BioSystems, Spain). TS% is presented as the quotient of serum iron and TIBC multiplied by 100 to obtain a percentage value.

In parallel, inflammatory markers were analyzed: interleukin-1 (IL-1), interleukin-6 (IL-6), TNF-*α*, interferon-*γ* (IFN-γ), serum amyloid A (SAA), haptoglobin (Hp) and extracellular Hsp70 (eHsp70). Sandwich ELISAs (Cloud Clone Corp., People’s Republic of China) were used, according to the manufacturer’s instructions. All samples were analyzed simultaneously and under the same laboratory conditions to minimize analytical variability. A microreader RT-2100C (Rayto, People’s Republic of China) was used for reading. The assays used had intra-assay CV < 10% and inter-assay CV < 12%. The minimum detectable dose is typically <6.1 pg/mL for IL-1, <3.1 pg/mL for IL-6, <3.1 pg/mL for TNF-*α*, <5.7 pg/mL for IFN-*γ*, <5.9 ng/mL for Hp, <2.72 ng/mL for SAA and <1.31 ng/mL for eHsp70.

### 2.3 Descriptive and longitudinal analysis

The aim of the longitudinal analysis was to characterize the temporal dynamics of all investigated parameters during early lactation and to identify distinct phases of the physiological response following calving.

Repeated measurements were analyzed using the following linear model:

$$Y_{\mathrm{ij}}=\mu +\mathrm{Tim}e_{j}+\mathrm{Co}w_{i}+\varepsilon_{\mathrm{ij}}$$

where *Y_ij_* represents the value of a given parameter for cow *i* at time point *j*, *μ* is the overall mean, *Time_j_* is the fixed effect of time, *Cow_i_* is the individual effect of cow, and *ε_ij_* is the residual error.

### 2.4 Heat map and cluster analysis

The aim of the heat map and cluster analysis was to identify global patterns of association among biomarkers and time points, as well as to visualize coordinated biological dynamics between inflammatory and iron metabolism parameters.

To ensure comparability among variables measured on different scales, all data were standardized using z-score transformation. Similarity between samples was quantified using Euclidean distance:

$$d(x,y)=\sqrt{\sum \begin{array}{c} p\\ i=1 \end{array}{\left(\mathrm{Xi}-\mathrm{Yi}\right)}^{2}}$$

where *x* is the first point of the coordinate, *y* is the second point of the coordinate, *x-y* the difference of the coordinates in each dimension.

Hierarchical clustering was performed using the Ward.D2 method, which minimizes within-cluster variance. Heat map was used for simultaneous visualization of temporal clusters (Day 0–21), grouping of cows according to response patterns and biomarker clusters according to functional similarity.

### 2.5 Correlation between examined parameters and multicollinearity

Pearson’s correlation coefficient was used to quantify the relationship between the examined parameters. The analysis assumed linearity of the relationship, approximate normality and homogeneity of variances. Multicollinearity analysis was then performed in order to analyze the mutual dependence of inflammatory biomarkers and validate their suitability for joint inclusion in regression models. Multicollinearity was assessed using the variance inflation factor (VIF) as well as the inversion of the correlation matrix:

$$\mathrm{VI}F_{j}=\frac{1}{1-R_{j}^{2}}$$

$$\mathrm{VIF}=\mathrm{diag}(R^{-1})$$

where *R_j_^2^* is the coefficient of determination obtained by regressing variable *Xj* on all other independent variables; *(R^−1^)_jj_* diagonal element of the inverse correlation matrix for variable *j*.

A threshold of VIF > 5 was considered indicative of substantial multicollinearity, suggesting shared variance among inflammatory markers.

### 2.6 Analysis of the principal components and the score value of the first principal inflammatory component during the experimental period

Principal Component Analysis (PCA) was applied with the aim of reducing the dimensionality of the data and solving the problem of pronounced multicollinearity between inflammatory biomarkers. Correlation analysis and VIF showed that inflammatory parameters shared significant common variance, indicating that they do not represent independent biological processes, but different manifestations of a unique systemic inflammatory response. Therefore, PCA was used as a method that enables the transformation of the original mutually correlated variables into a smaller number of mutually orthogonal (uncorrelated) components, while preserving most of the total variability of the data. Before performing PCA, all variables were standardized by z-score transformation in order to eliminate the influence of different measurement scales and allow equal contribution of all biomarkers in the analysis. For principal component analysis (PCA), the number of retained components was determined based on the proportion of explained variance, inspection of the scree plot, and the Kaiser criterion (eigenvalues > 1). Components were retained when they contributed substantially to the total variance and improved the interpretability of the multivariate structure. The adequacy of PCA was assessed using the Kaiser–Meyer–Olkin (KMO) measure of sampling adequacy and Bartlett’s test of sphericity.

Mathematically, PCA transforms the original variables into linear combinations:

$$Z_{k}=a_{k1}X_{1}+a_{k2}X_{2}+\ldots +a_{\mathrm{kp}}X_{p}$$

where *Zk* represents the main component, X_p_ the original standardized variables, while a_kp_ represent weight coefficients (loadings) that define the contribution of each variable to an individual component.

The first principal inflammatory component PC1-infla is described by formula:

$$\mathrm{PC}1-\text{infla}=\sum \begin{array}{c} p\\ j=1 \end{array}a_{1j}X_{j}$$

where PC1 represents a linear combination of all inflammatory biomarkers *X* that explain the largest part of the total variance in the data, p represents the total number of inflammatory markers included in the PCA, and a_1j_ represents the loading coefficient that defines the contribution of each individual variable to the first principal component.

After conducting PCA, each observed cow was assigned principal component scores marked as PC1-infla. These values represent the projection of the original data onto the new orthogonal dimensions defined by the PCA, with PC1-infla explaining most of the total variance. Consequently, each experimental cow received a unique numerical PC1-infla score, which was subsequently used in statistical analyses as a reduced representation of the original set of variables. PC1-infla was interpreted as a latent indicator of the inflammatory response pattern. The effect of time on PC1-infla scores was evaluated using a linear model, following the same approach applied to the individual parameters. Temporal changes in PC1-infla were then visualized graphically for each cow separately.

### 2.7 Linear mixed models in the assessment of the influence of inflammatory parameters on iron parameters

The goal of applying linear mixed models was to quantify the effect of systemic inflammation on iron metabolism, controlling for temporal changes and individual differences between cows. For this purpose, a univariate model was used with PC1-infla as an independent variable affecting iron parameters. Cows were included as a random effect to account for within-subject correlation among repeated measurements. For linear mixed-effects models, model assumptions were evaluated through standard diagnostic procedures. The normality of residuals was assessed using quantile–quantile (Q–Q) plots and inspection of residual distributions, while homoscedasticity was evaluated by examining residuals versus fitted values plots. Potential influential observations were assessed using residual diagnostics. Model convergence and the appropriateness of the random-effects structure were also checked. No major deviations from model assumptions were observed.

The following formulas were used:

$$Y_{\mathrm{ij}}=\beta_{0}+\beta_{1}\mathrm{PC}1_{\mathrm{ij}}+\beta_{2}\mathrm{Tim}e_{j}+u_{i}+\varepsilon_{\mathrm{ij}}$$

where Y*ij* is parameter of iron metabolism (Fe, TIBC, UIBC, TS%), *β0* intercept, *β_1_* is the effect of PC1-infla score, *β_2_Time*- influence of time, *u_i_* is random intercept for cow and *ε_ij_* residual error.

In addition to the above, two supplementary models (univariate and multivariate) where the independent variables were inflammatory markers and not the PC1-infla component were determined. The following models were used:

(i) Univariate model – influence of individual inflammatory parameters on iron parameters according to the formula:

$$Y_{\mathrm{ij}}=\beta_{0}+\beta_{1}X_{\mathrm{ij}}^{\left(k\right)}+\beta_{2}\mathrm{Tim}e_{j}+u_{i}+\varepsilon_{\mathrm{ij}}$$

where Y*ij* is parameter of iron metabolism (Fe, TIBC, UIBC, TS%), *β0* intercept, $\beta_{1}x_{ij}^{\left(k\right)}\;$ individual inflammatory biomarker, *β_2_ Time* influence of time, *u_i_* is random intercept for cow and *ε_ij_* residual error.

(ii) Multivariate linear model – group influence of all inflammatory parameters on iron parameters according to the formula:

$$\begin{array}{c} Y_{\mathrm{ij}}=\beta_{0}+\beta_{1}\mathrm{IL}1_{\mathrm{ij}}+\beta_{2}\mathrm{IL}6_{\mathrm{ij}}+\beta_{3}\mathrm{TNF}\alpha_{\mathrm{ij}}+\beta_{4}\mathrm{IFN}\gamma_{\mathrm{ij}}+\beta_{5}\mathrm{SA}A_{\mathrm{ij}}\\ +\beta_{6}Hp_{\mathrm{ij}}+\beta_{7}\text{eHSP}70_{\mathrm{ij}}+\beta_{8}\mathrm{Tim}e_{j}+u_{i}+\varepsilon_{\mathrm{ij}} \end{array}$$

where Y*ij* is parameter of iron metabolism (Fe, TIBC, UIBC, TS%), *β_1_* to *β_7_* is inflammatory biomarker effect, *β_8_Time* influence of time, *u_i_* is random intercept for cow and *ε_ij_* residual error.

In univariate models, each inflammatory parameter individually demonstrates an association with the outcome because, in addition to its own effect, it partially captures the common inflammatory signal shared with other highly correlated biomarkers. In contrast, in multivariate models, this shared information is statistically distributed across several interrelated parameters, causing individual regression coefficients to become unstable or lose statistical significance. Therefore, these models should not be interpreted as estimates of the independent effects of individual biomarkers, but rather as evidence supporting the existence of a common inflammatory pattern represented by the PC1-infla component.

Although the model incorporating the PC1-infla component was considered the primary analytical approach due to the pronounced multicollinearity among inflammatory parameters, univariate and multivariate models were additionally performed to facilitate interpretation and evaluate the robustness of the findings.

The univariate models were used to assess whether individual inflammatory parameters consistently reflected the same underlying inflammatory signal captured by the PC1-infla component. Conversely, the multivariate models were used to determine whether the observed association persisted after the simultaneous inclusion of several correlated biomarkers, thereby evaluating whether the finding reflected a common inflammatory pattern rather than the influence of a single dominant marker. Given the high degree of intercorrelation among the inflammatory parameters, the results of these supplementary analyses were interpreted primarily as confirmation of the consistency and stability of the main finding obtained using the PC1-infla component.

Parity was initially considered as a biologically relevant explanatory variable. However, because it did not significantly improve model performance, it was excluded from the final models to maintain model parsimony. Interaction terms among inflammatory biomarkers were not included because of the high degree of collinearity among the biomarkers, which was addressed through principal component analysis.

Statistical analyses were conducted using RStudio (version 2025.05.1; R Foundation for Statistical Computing, Austria) with the packages *lme4* for mixed-effects modeling, *lmerTest* for significance testing, *FactoMineR* and *factoextra* for principal component analysis, and the *stats* package for correlation analyses. Additional statistical analyses were performed using SPSS Statistics (version 29; IBM Corp., USA).

## 3 Results

### 3.1 Changes in inflammatory parameters and iron parameters in early lactation

To assess iron status and the inflammatory response in dairy cows, changes in the concentrations of the analyzed parameters were monitored throughout the experimental period (Figure 1). The results demonstrated a marked reduction in Fe concentration, TIBC, UIBC, and TS% during early lactation. Serum Fe concentration and TS% began to decline immediately after calving and were significantly lower on Days 7 and 14 postpartum, followed by a gradual recovery. The lowest values for both parameters were observed on Day 7. TIBC and UIBC exhibited a more moderate decline during the first days after calving, reaching their lowest values on Day 7, after which both parameters progressively increased. Inflammatory cytokines displayed a pronounced increase during the early postpartum period. Concentrations of IL-1, IL-6, TNF-*α*, and IFN-*γ* reached their peak values between Days 3 and 7, followed by a gradual decline toward baseline levels by Day 21. The most pronounced responses were observed for IL-6 and IFN-γ, which exhibited the largest increases relative to their initial concentrations. Acute-phase proteins also showed substantial temporal variation. SAA increased sharply by Day 3 and subsequently declined throughout the remainder of the experimental period. In contrast, Hp increased progressively, reaching its maximum concentration on Day 7 before gradually decreasing to its lowest values by Day 21. The concentration of eHsp70 exhibited a moderate increase during the early and middle phases of the experiment, with peak values observed between Days 3 and 14, followed by a decline toward the end of the study.

**Figure 1:**
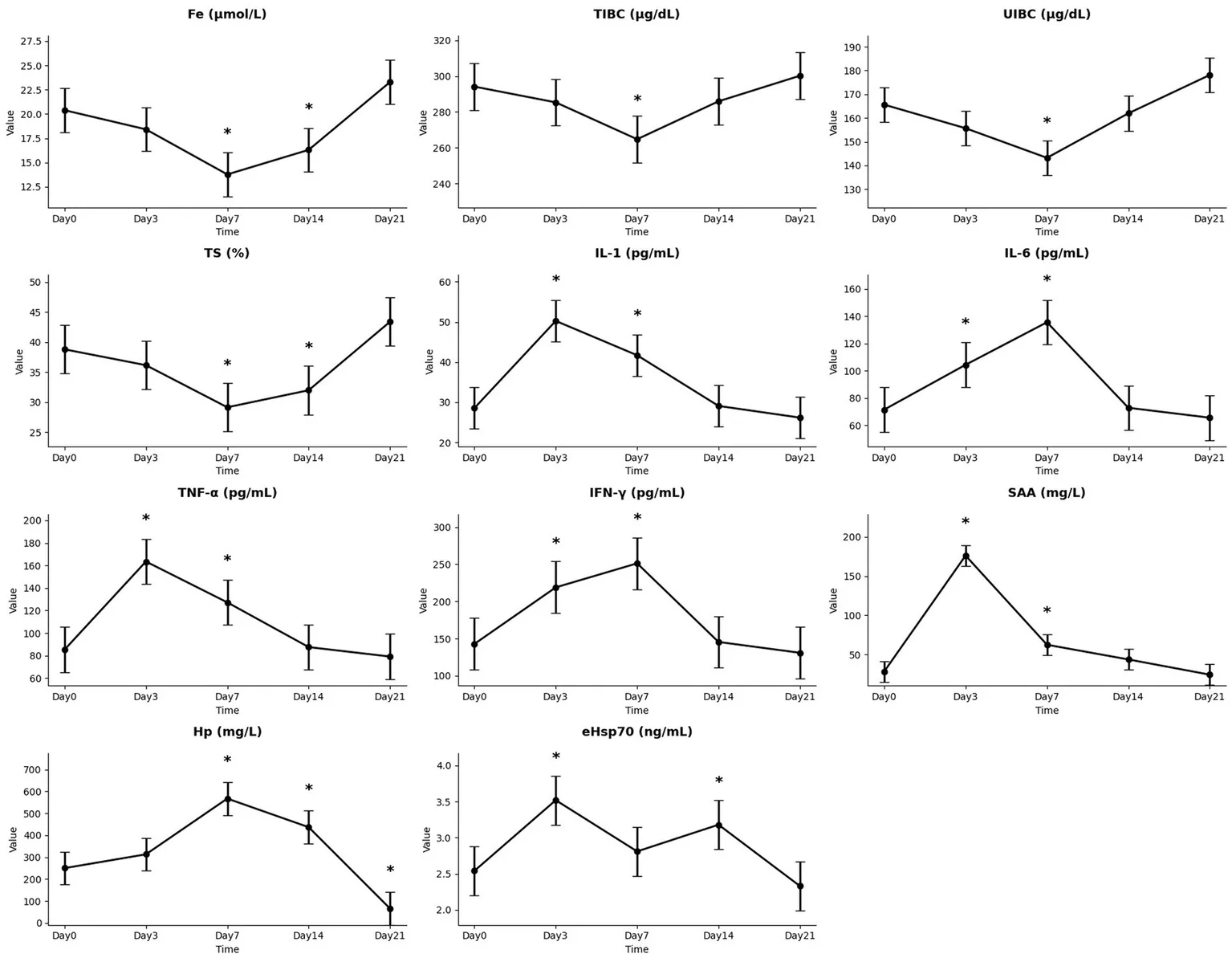
Influence of time on the value of the examined parameters. Iron-related parameters declined during early lactation, reaching minimum values around Day 7, followed by partial recovery by Day 21. In contrast, inflammatory markers exhibited a transient increase, peaking between Days 3 and 7 before gradually returning toward baseline.

### 3.2 Global expression patterns of the examined parameters

The heat map shows the global patterns of the study data across each individual cow in the trial, time and clusters (Figure 2). Temporal clustering on the heat map shows a clear separation of individuals in different experimental days according to the intensity of the inflammatory response and changes in iron metabolism: the closest clusters are day 3 and day 7, the next important cluster is days 0 and 21 where individual cows are mixed, and the day 14 cluster is closer to the day 0 and day 21 cluster. The heat map enables an overview of the dynamic changes of biomarkers in individual cows over time. Two groups of clusters were formed, namely the cluster of iron parameters and the cluster of inflammatory parameters. The cluster of iron parameters confirms the same pattern of change of Fe, TIBC, UIBC, TS%, with the lowest values on Day 7. When it comes to the inflammatory cluster, IL-6, IFN-*γ* and Hp, which show the highest expression on the third day, then IL-1, TNF-*α*, SAA and eHsp70, which have the highest expression on the seventh day, can be clearly distinguished. The heat map shows that with an increase in the expression of inflammatory parameters, there is a decrease in the expression of iron parameters, and almost all individuals in the test have this metabolic imprint.

**Figure 2:**
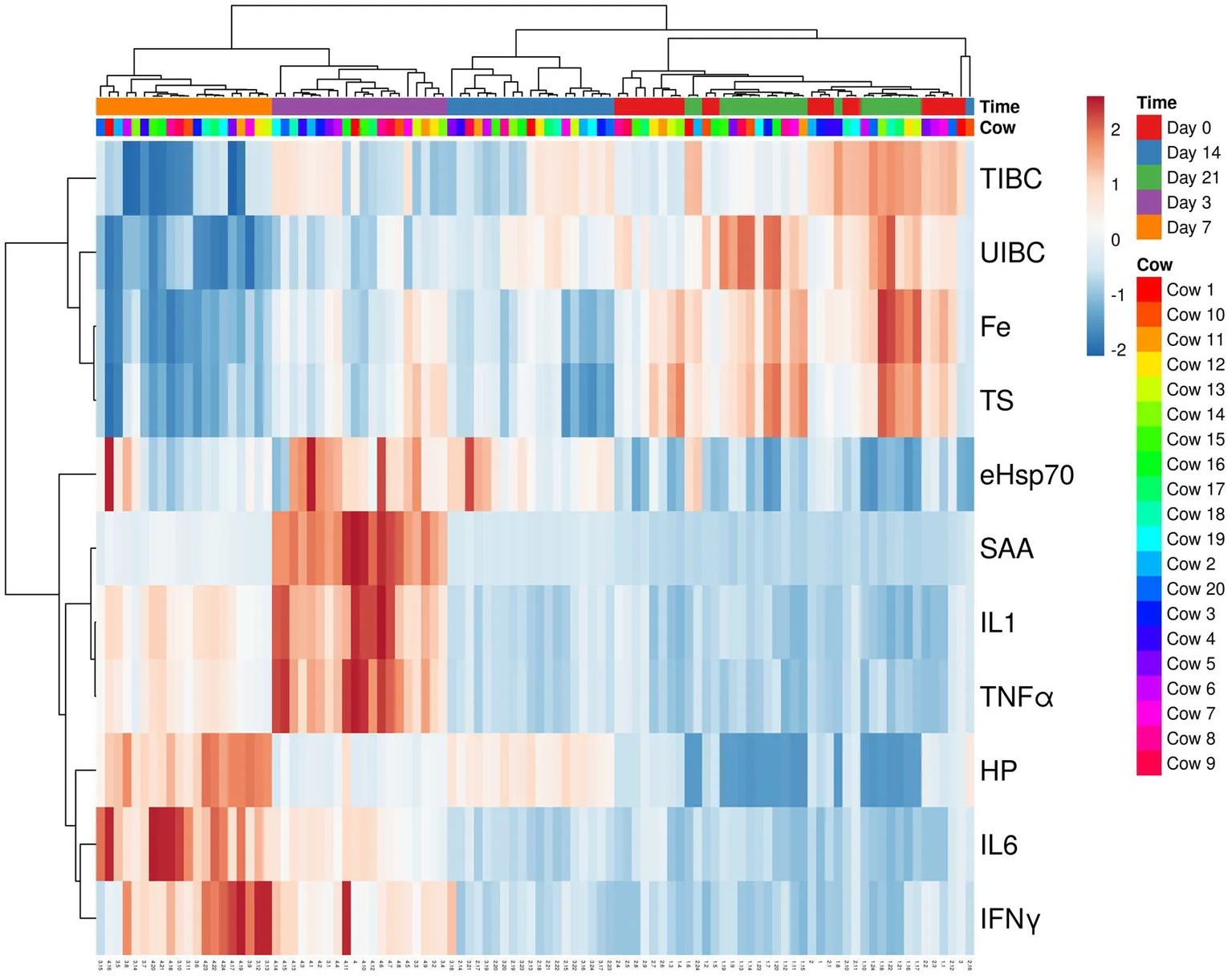
Heat map and cluster analysis show global patterns of association among biomarkers and time points. Multivariate clustering and heatmap analyses revealed a clear temporal separation between the inflammatory activation phase and the recovery phase, indicating an inverse relationship between inflammatory status and iron bioavailability.

### 3.3 Correlation analysis of inflammatory and iron parameters

Following visual assessment of biomarker patterns using the heat map, correlation analysis was performed to quantify the relationships among the analyzed parameters. The results are presented in the form of a correlation matrix (Figure 3). Parameters of iron metabolism exhibited consistent negative correlations with inflammatory biomarkers, indicating a strong association between inflammatory activation and alterations in iron homeostasis. Serum iron (Fe) showed the strongest inverse relationships with Hp (*r* = −0.827; very strong correlation) and IL-6 (*r* = −0.702; strong correlation), as well as moderate negative correlations with IFN-*γ* (*r* = −0.526), IL-1 (*r* = −0.469), and TNF-*α* (*r* = −0.411). These findings suggest a substantial influence of inflammatory processes on the reduction of circulating iron concentrations. TIBC exhibited moderate to strong negative correlations with IL-6 (*r* = −0.605), Hp (*r* = −0.599), and IFN-*γ* (*r* = −0.524), indicating a consistent response to inflammatory activation. UIBC demonstrated particularly strong negative correlations with both IL-6 and Hp (*r* = −0.790 for both), as well as with IFN-γ (*r* = −0.701). Moderate negative correlations were also observed with IL-1 (*r* = −0.592) and TNF-*α* (*r* = −0.549), highlighting the high sensitivity of UIBC to inflammatory changes. Similarly, TS% showed pronounced negative associations, particularly with Hp (*r* = −0.771) and IL-6 (*r* = −0.640), and moderate negative correlations with IFN-*γ* (*r* = −0.448) and IL-1 (*r* = −0.423), confirming the reduction in transferrin saturation during inflammatory activation. In contrast, eHsp70 exhibited only weak negative correlations with all indicators of iron status.

**Figure 3:**
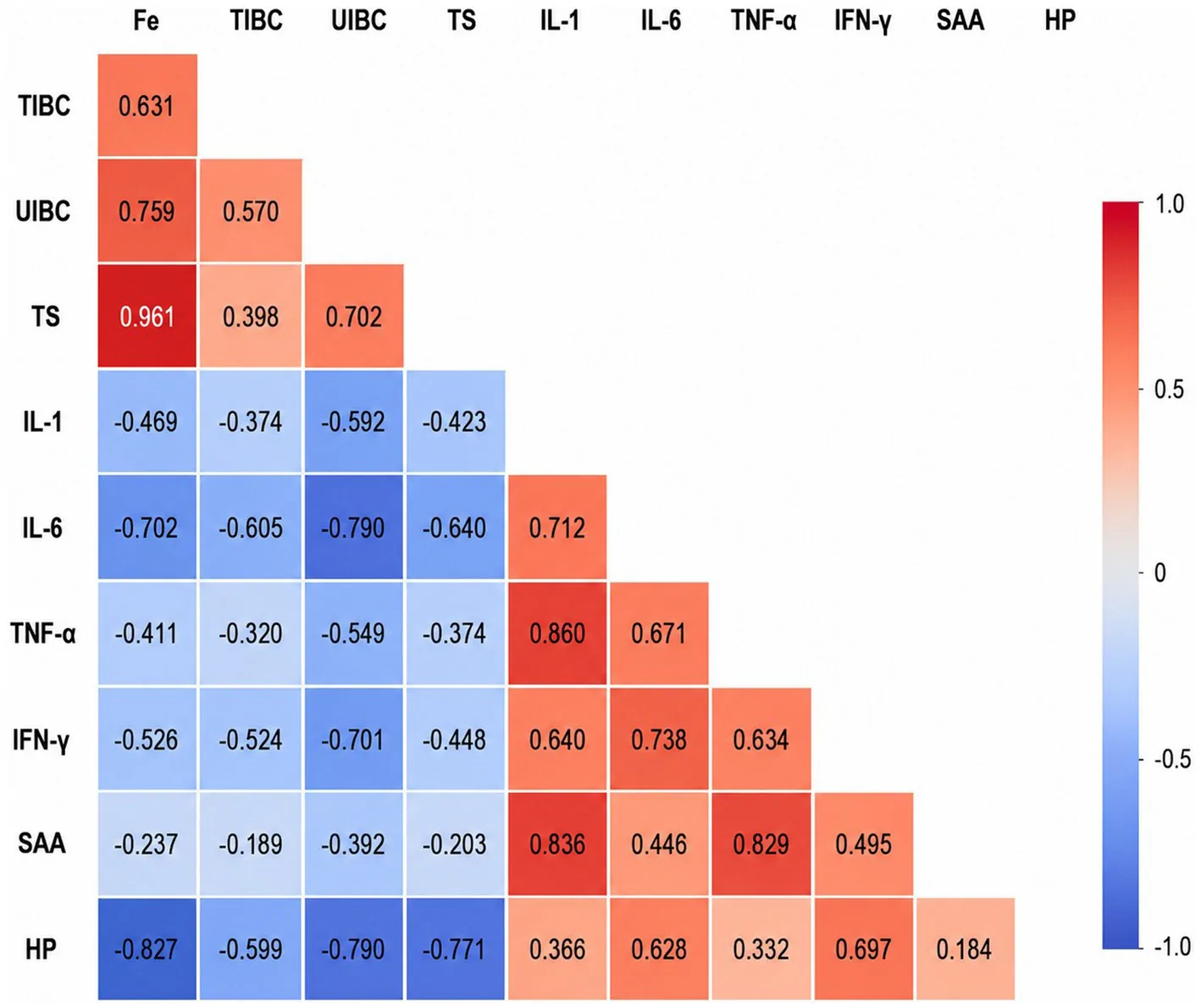
Correlation coefficient matrix between parameters. Correlation analysis confirmed significant negative associations between inflammatory mediators, particularly IL-6 and haptoglobin, and all iron metabolism indicators. The correlation is significant at the *p* < 0.05 level if |*r*| ≥ 0.20, and at the *p* < 0.01 level if |*r*| ≥ 0.26.

Inflammatory cytokines form a highly coherent network with pronounced positive correlations. The strongest association was observed between IL-1 and TNF-*α* (*r* = 0.860), followed by IL-1 and IL-6 (*r* = 0.712), and IL-6 and TNF-*α* (*r* = 0.671), underscoring the central role of these cytokines in coordinating the inflammatory response. IFN-*γ* was also strongly integrated within this network, exhibiting strong positive correlations with IL-6 (*r* = 0.738), IL-1 (*r* = 0.640), and TNF-*α* (*r* = 0.634), suggesting an important role in amplifying inflammatory signaling pathways. Among the acute-phase proteins, SAA displayed very strong positive correlations with IL-1 (*r* = 0.836) and TNF-α (*r* = 0.829), as well as moderate positive correlations with IFN-*γ* (*r* = 0.495) and IL-6 (*r* = 0.446), identifying it as one of the biomarkers most closely associated with acute inflammatory activity. Hp shows strong correlations with IFN-γ (*r* = 0.697) and IL-6 (*r* = 0.628). eHsp70 shows only weak positive correlations with inflammatory response parameters.

### 3.4 Modeling the effect of inflammatory parameters on parameters of iron metabolism and evaluation of latent index of inflammation

The effects of inflammatory parameters on iron metabolism were evaluated using linear mixed-effects models (LMMs), with cow included as a random effect and day as a fixed effect. This approach enabled the estimation of the effects of inflammatory biomarkers on iron-related parameters independently of the physiological changes in iron metabolism that occur throughout early lactation. Both univariate models, assessing the effects of individual inflammatory parameters, and multivariate models, including all inflammatory biomarkers simultaneously, were considered.

An important prerequisite for multivariate LMM analysis is the absence of substantial multicollinearity among the independent variables. The analysis revealed pronounced collinearity within the inflammatory biomarker dataset. High VIF values were observed for IL-1 (6.70), SAA (6.07), and TNF-*α* (5.43), indicating substantial multicollinearity according to conventional criteria (VIF > 5). Moderate collinearity was detected for IL-6 (4.01) and IFN-*γ* (3.60), whereas Hp (2.96) and eHsp70 (1.85) exhibited relatively low collinearity and could be considered more independent variables. These findings suggest the existence of a strongly interconnected subset of inflammatory biomarkers that likely reflects a common underlying biological process. The pronounced multicollinearity among inflammatory parameters indicates that these variables share a considerable proportion of variance and cannot be reliably separated into independent effects within a single regression model. Consequently, the inclusion of all inflammatory biomarkers in a multivariate model would likely result in unstable coefficient estimates, inflated standard errors, and reduced interpretability of individual regression coefficients (Supplementary Figure S1).

To address this issue, principal component analysis (PCA) was applied as a dimensionality-reduction technique. PCA transforms correlated variables into a smaller number of mutually orthogonal components while retaining most of the total variance contained in the original dataset. The first principal component (PC1-infla) explained 63.5% of the total variance, indicating that it captured the dominant shared signal among the inflammatory biomarkers. This component was therefore interpreted as a latent index of inflammatory activity and designated PC1-infla. The largest contributions to PC1-infla originated from IL-1, TNF-*α*, and SAA, indicating that the component primarily reflects a coordinated pro-inflammatory response. IL-6 and IFN-*γ* contributed moderately, whereas Hp and eHsp70 had relatively minor contributions to the component structure (Figure 4). These findings confirm that the inflammatory biomarkers do not act as independent variables but rather represent different manifestations of a common biological inflammatory process.

**Figure 4:**
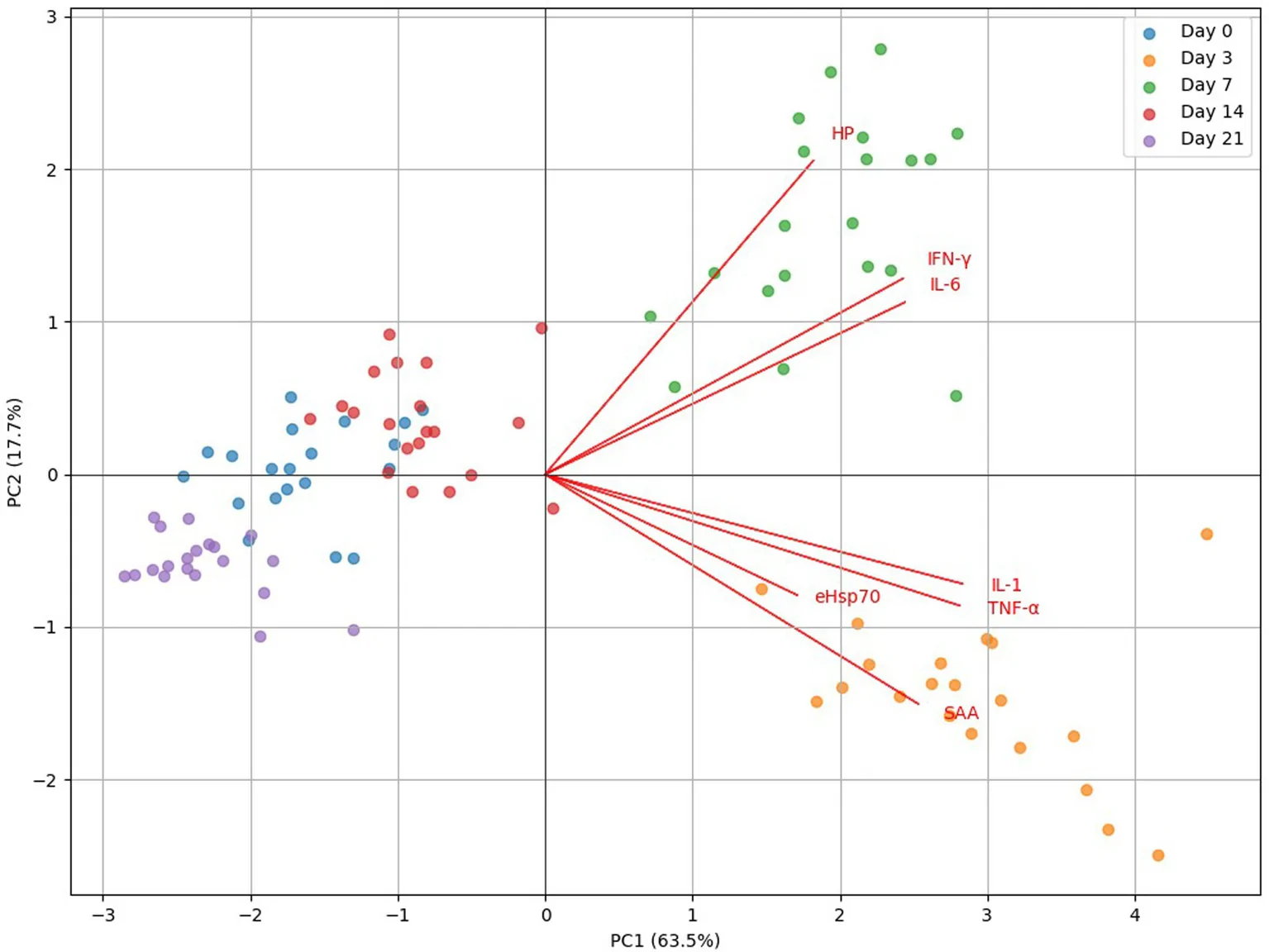
PCA composite index on inflammatory parameters. Because of substantial multicollinearity among inflammatory variables, PCA was applied to generate a latent inflammatory score. The first principal inflammatory component (PC1-infla) explained 63.5% of the total variance and represented overall systemic inflammatory activity.

PC1-infla scores exhibited pronounced temporal dynamics throughout the experimental period. Values were predominantly negative on Day 0, increased sharply to reach their maximum on Day 3, declined on Day 7, returned to approximately baseline levels by Day 14, and became negative again on Day 21. The consistency of this pattern across all animals indicates that PC1-infla captures a dominant biological response characteristic of early lactation. High positive PC1-infla scores on Day 3 reflected strong activation of the inflammatory biomarkers contributing positively to the first principal component, whereas the negative scores observed at the beginning and end of the experiment represented the opposite biomarker profile (Figure 5).

**Figure 5:**
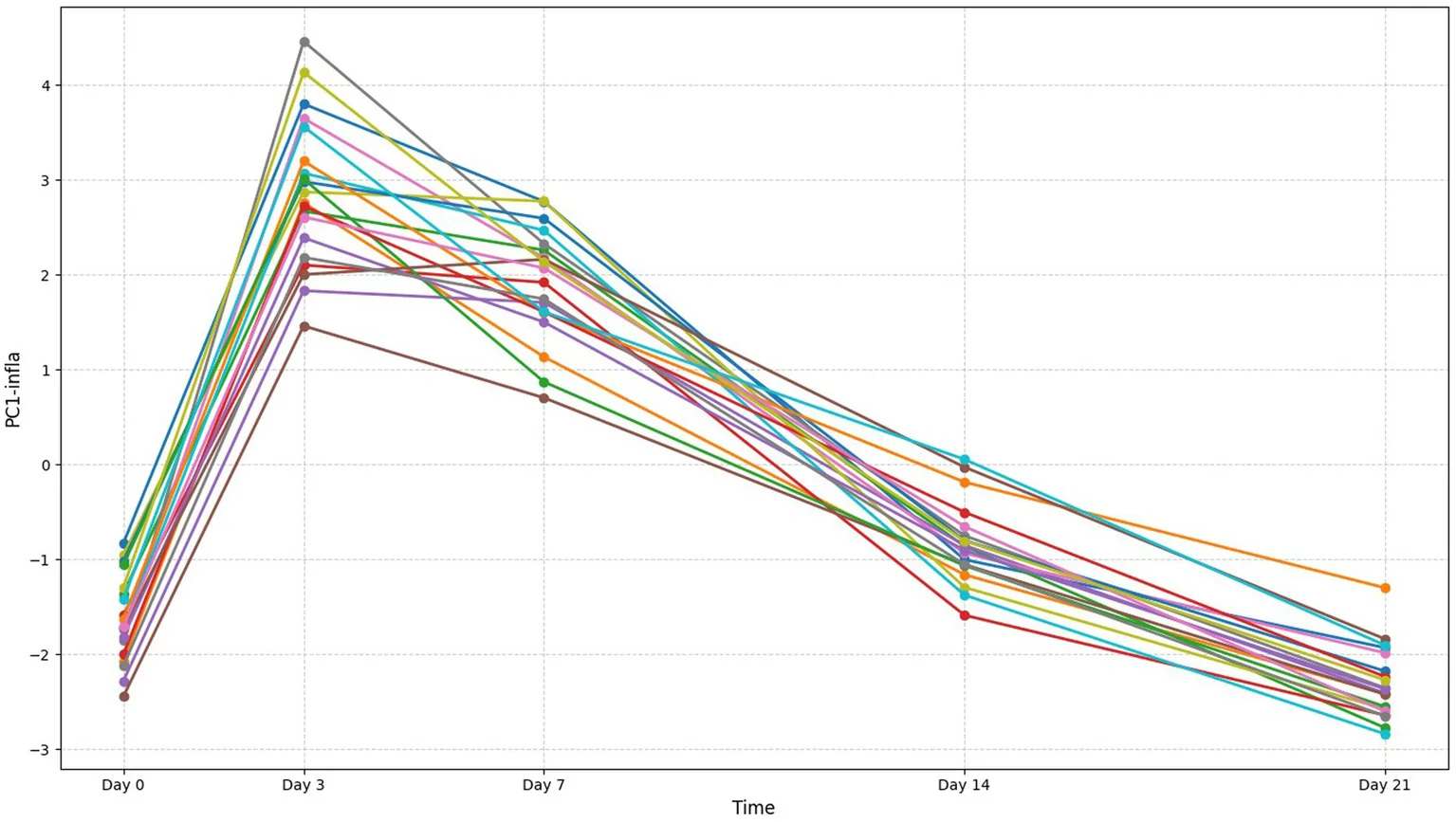
Cow-level PC1-infla scores during the experimental period. PC1-infla scores exhibited pronounced temporal dynamics throughout the experimental period. High positive PC1-infla scores on Day 3 reflected strong activation of the inflammatory biomarkers contributing positively to the first principal component, whereas the negative scores observed at the beginning and end of the experiment represented the opposite biomarker profile.

The association between PC1-infla and iron metabolism parameters was subsequently evaluated using linear mixed-effects models that included day as a fixed effect and a random intercept for each cow (CowID) to account for repeated measurements within animals. The results demonstrated statistically significant associations between PC1-infla and all analyzed indicators of iron metabolism (Table 1).

**Table 1:** Linear mixed model influence PC1-infla on iron blood parameters in early lactation when cow is random factor and day fixed factor in model.

Dependent variable	Model	Estimate	CI Lower	CI upper	Std. error	Statistic	*p*-value
Fe	(Intercept) PC1-infla	18.44 −1.09	17.85 −1.37	19.03 −0.81	0.3 0.14	61.37 −7.64	<0.001 <0.001
TIBC	(Intercept) PC1-infla	285.8 −4.13	281.69 −5.41	289.91 −2.85	2.1 0.65	136.36 −6.31	<0.001 <0.001
UIBC	(Intercept) PC1-infla	160.91 −4.58	159.22 −5.39	162.6 −3.77	0.86 0.41	186.17 −11.11	<0.001 <0.001
TS%	(Intercept) PC1-infla	35.89 −1.62	34.87 −2.11	36.91 −1.14	0.52 0.25	68.99 −6.54	<0.001 <0.001

Linear mixed-effects models demonstrated that higher PC1-infla scores were significantly associated with lower Fe, TIBC, UIBC, and TS% values (*p* < 0.001). Compared with individual biomarkers, PC1-infla provided a more robust and biologically meaningful associated with of iron bioavailability by integrating the shared variance of the inflammatory network.

For serum iron (Fe), higher PC1-infla scores were associated with significantly lower Fe concentrations (*β* = −1.09; 95% CI: −1.37 to −0.81; *p* < 0.001). A similar relationship was observed for total iron-binding capacity (TIBC), where increased inflammatory activity was associated with lower TIBC values (*β* = −4.13; 95% CI: −5.41 to −2.85; *p* < 0.001). Likewise, higher PC1-infla scores were associated with significantly lower unsaturated iron-binding capacity (UIBC) values (*β* = −4.58; 95% CI: −5.39 to −3.77; *p* < 0.001). For transferrin saturation (TS%), increasing inflammatory burden was associated with a significant reduction in TS% (*β* = −1.62; 95% CI: −2.11 to −1.14; *p* < 0.001).

Supplementary Table S1 show that individual inflammatory parameters have the same direction of action as PC1-infla. The change in the direction of action of some of the inflammatory parameters in multivariate models occurs as a consequence of the statistical tendency of the model to take away the joint effect of one of the parameters and attribute it to another, which is a consequence of multicollinearity, and not a change in the biological effect of the parameter. Supplementary models confirmed the significance of the PC1-infla integrated inflammatory score in assessing iron status in early lactation.

Graphical analyses further confirmed the biological significance of PC1-infla, as negative associations between PC1-infla and all investigated iron-related parameters were consistently observed in each individual cow included in the study (Figure 6). This finding suggests the existence of a stable and biologically meaningful relationship between inflammatory status and iron metabolism, independent of individual differences in the absolute values of the measured parameters. Although cows differed in their baseline values and in the magnitude of their responses, the direction of the association remained consistently negative across all animals. This result indicates that PC1-infla represents a robust latent indicator of systemic inflammation that is consistently associated with a reduction in iron availability and transport. Overall, the findings suggest that inflammation has a dominant suppressive effect on parameters of iron metabolism, with the effect being consistent across all observed individuals.

**Figure 6:**
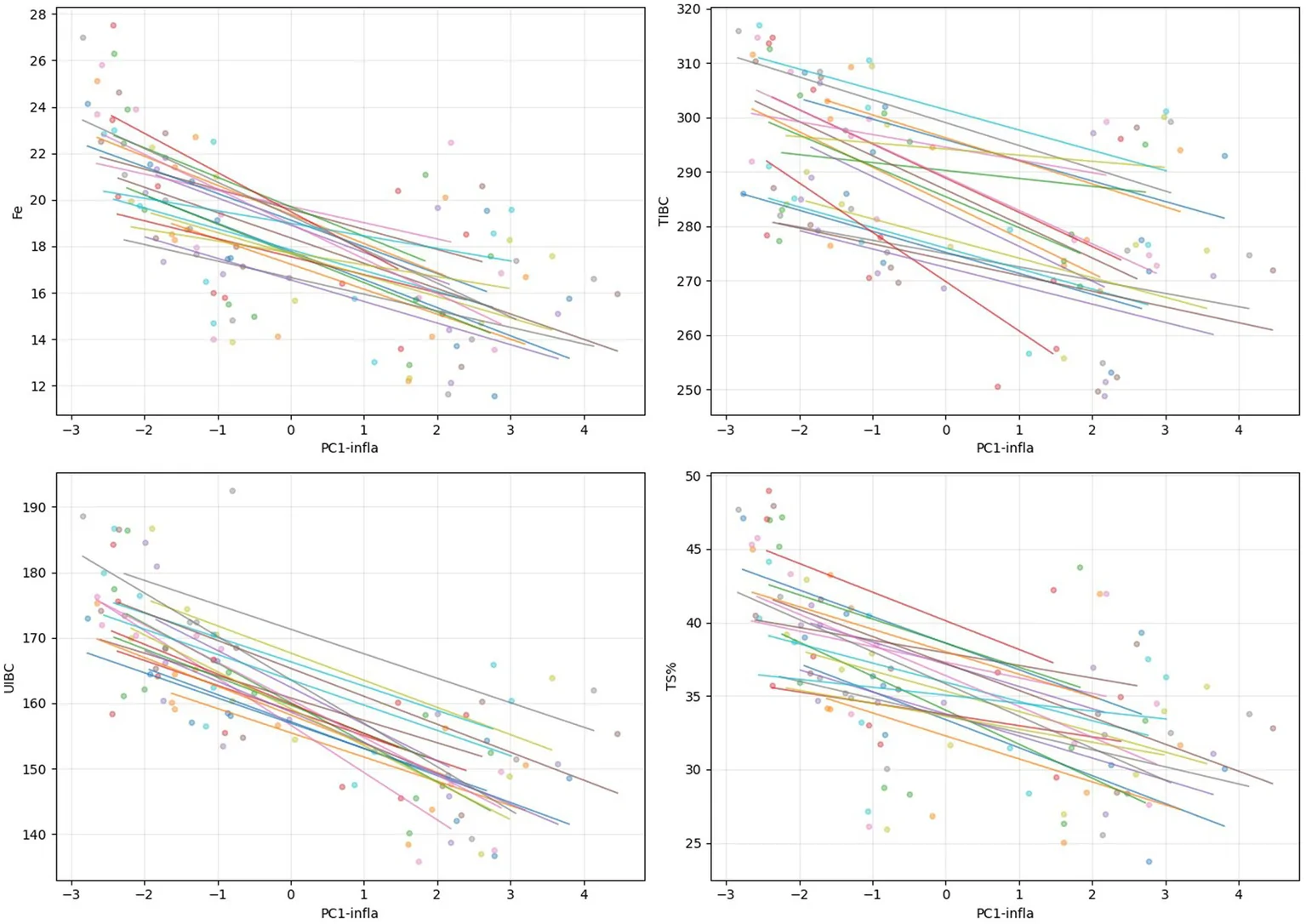
Cow-level regression of PC1-infla against iron metabolism markers over time. Graphical analyses further confirmed the biological significance of PC1-infla, as negative associations between PC1-infla and all investigated iron-related parameters were consistently observed in each individual cow included in the study.

## 4 Discussion

### 4.1 Changes in inflammatory parameters and Fe parameters in early lactation

Traditionally, negative energy balance has been regarded as the primary physiological challenge of the transition period in dairy cows, whereas inflammation has largely been viewed as a secondary consequence of metabolic adaptation. However, contemporary research suggests that metabolic and inflammatory changes are closely interconnected components of a coordinated adaptive process that facilitates the redistribution of nutrients and energy toward the mammary gland following calving (4–6, 31). Consequently, inflammation is no longer considered solely an undesirable phenomenon but rather a physiological regulatory mechanism that enables the organism to adapt to the substantial metabolic demands associated with the onset of lactation.

In the present study, concentrations of IL-1, IL-6, TNF-*α*, and IFN-*γ* exhibited highly similar temporal patterns, reaching their highest values between the third and seventh day postpartum. This observation is consistent with previous reports indicating that parturition is followed by activation of tissue macrophages in adipose tissue, the liver, and the reproductive tract, resulting in increased production of pro-inflammatory cytokines (2, 3, 8). Notably, IL-6 and IFN-*γ* exhibited the greatest relative increases compared with baseline concentrations. IL-6 is recognized as a central mediator of the acute-phase response in cattle because it links local inflammatory events with systemic metabolic adaptations through its action on hepatocytes and the subsequent induction of acute-phase protein synthesis (10, 11). In contrast, the increase in IFN-γ suggests that, in addition to classical inflammatory pathways, activation of the cellular immune response also occurs during the physiological adaptation to early lactation, as previously described (13).

The changes observed in acute-phase proteins further support the existence of a coordinated inflammatory response. Serum amyloid A (SAA) exhibited the most rapid response, reaching peak concentrations on the third day postpartum, whereas haptoglobin (Hp) peaked on the seventh day. This sequential pattern is fully consistent with the established biology of the acute-phase response in cattle. SAA is among the most sensitive inflammatory biomarkers, with concentrations capable of increasing several hundred-fold within hours of cytokine cascade activation, whereas Hp displays a slower but more sustained response and remains elevated in circulation for a longer period (12, 32). Similar temporal patterns have been reported in healthy transition cows in several independent studies, indicating that increased SAA and Hp concentrations do not necessarily reflect disease but may instead represent normal physiological postpartum adaptation (4, 6).

Although cytokine activation begins before calving, previous studies have demonstrated that low-grade inflammation persists during the postpartum period (33), which is consistent with our findings. A recent study reported increased TNF-*α* concentrations during early lactation, with a peak occurring during the first week postpartum, whereas IFN-*γ* concentrations were initially lower after calving and increased thereafter, partially differing from the pattern observed in the present study (34). Persistent low-grade inflammation during the first weeks postpartum has been extensively documented and is associated with insulin resistance, adipose tissue mobilization, and milk production. It also plays an important role in uterine involution, which involves remodeling of the cervix, fetal membranes, and myometrium, as well as in pathogen clearance, a critical mechanism for maintaining uterine and mammary gland health (35, 36). In healthy cows, higher Hp concentrations during the first weeks postpartum have been associated with more pronounced metabolic and inflammatory adaptations (37). Furthermore, administration of non-steroidal anti-inflammatory drugs has been shown to accelerate the decline of cytokines and acute-phase proteins by modulating inflammatory pathways and metabolic processes (38).

Unlike the classical inflammatory biomarkers, extracellular heat shock protein 70 (eHsp70) exhibited only a moderate increase during the early stages of the study and showed substantially weaker correlations with the other inflammatory parameters. This finding suggests that eHsp70 reflects a distinct aspect of transition-period adaptation. Extracellular Hsp70 is known to function as a damage-associated molecular pattern (DAMP), signaling cellular stress and tissue injury while modulating cytokine production and immune-cell activation (14). However, unlike IL-1, IL-6, or TNF-*α*, its concentration does not directly reflect the intensity of systemic inflammation. Instead, it appears to be influenced by both cellular stress and the activation of protective and adaptive mechanisms (39). The relatively weak correlations observed between eHsp70 and the other inflammatory biomarkers in the present study support this interpretation.

Parameters of iron bioavailability changed markedly during early lactation. All analyzed indicators exhibited a highly similar temporal pattern, reaching their lowest values on the seventh day postpartum, corresponding to the period of greatest inflammatory activity. This close synchronization strongly suggests that iron metabolism is not merely a passive consequence of metabolic stress but rather an actively regulated component of the host immune response, consistent with previous findings (40, 41). Most previous studies investigating the relationship between inflammation and iron metabolism have been observational in nature and conducted in animals affected by mastitis, metritis, pneumonia, or other clinically apparent inflammatory disorders; consequently, they have primarily reported associations rather than establishing direct causal relationships (16, 23). The results of those studies are largely consistent with the findings of the present investigation. The controlled reduction of circulating iron concentrations during inflammation, commonly referred to as inflammatory hypoferremia, has been documented in numerous animal species as well as in humans (18, 20). Present observation of similar changes in clinically healthy cows during early lactation supports the hypothesis that low-grade physiological inflammation can substantially influence iron bioavailability and iron transport parameters (42, 43), which are sensitive indicators of inflammatory activity.

Although parity has been reported to influence metabolic adaptation during early lactation, no significant effect was observed in the present study, possibly because only second- and third-lactation cows were included, resulting in a relatively homogeneous study population.

### 4.2 Global expression patterns of investigated parameters and correlation analysis

Additional evidence supporting the close relationship between the inflammatory response and iron metabolism was provided by the heat map and correlation analyses. Correlation analysis further quantified these associations. The strongest negative correlations were observed between Fe and Hp (*r* = −0.827), Fe and IL-6 (*r* = −0.702), and between UIBC and both Hp and IL-6 (*r* = −0.790). The magnitude of these correlations suggests that IL-6 and Hp most accurately reflect the intensity of changes in iron bioavailability during early lactation. This finding is not unexpected given the central role of IL-6 in coordinating the acute-phase response.

Across mammalian species, IL-6 serves as a key mediator linking activation of the innate immune system with alterations in mineral metabolism, particularly iron metabolism (17, 18). A central component of this process is hepcidin, a peptide hormone synthesized primarily in the liver and recognized as the principal regulator of systemic iron homeostasis (17). During inflammation, IL-6 stimulates hepcidin expression in hepatocytes through activation of the JAK/STAT3 signaling pathway. Increased hepcidin concentrations promote the internalization and degradation of ferroportin, the only known cellular iron exporter responsible for releasing iron from enterocytes, macrophages, and hepatocytes into the circulation. As a result, iron release into plasma is reduced, leading to decreased serum Fe concentrations and lower transferrin saturation (17, 18).

Simultaneously, TNF-*α* and IL-1 may suppress transferrin synthesis, as transferrin is a negative acute-phase protein, thereby contributing to reductions in TIBC and UIBC values (19, 20). Although hepcidin was not measured in the present study, which may be considered a limitation, several indirect lines of evidence support the involvement of this mechanism. First, peak IL-6 concentrations preceded or coincided with the lowest values of Fe and TS%. Second, IL-6 exhibited some of the strongest negative correlations with all analyzed indicators of iron bioavailability. Third, the observed temporal patterns were highly consistent across individual animals. Based on these observations and the established physiological mechanisms regulating iron homeostasis, it is reasonable to propose that inflammation-induced activation of the hepcidin pathway was a major contributor to the alterations in iron metabolism observed during the study period.

Interestingly, Hp exhibited even stronger negative correlations with iron-related parameters than IL-6. This likely reflects the fact that Hp integrates the cumulative effects of multiple inflammatory signaling pathways. Whereas cytokine concentrations often fluctuate rapidly and transiently, Hp remains elevated for a longer duration and may therefore serve as a more stable indicator of the overall inflammatory burden. Similar findings have been reported by Tsukano and colleagues, who demonstrated that serum iron concentrations in cattle were more strongly associated with acute-phase proteins than with individual cytokines (15, 16).

In contrast to the other inflammatory biomarkers, eHsp70 showed only weak correlations with both iron-related parameters and inflammatory cytokines. This observation suggests that eHsp70 reflects a distinct aspect of the biological response, potentially related to cellular stress, tissue damage, and cytoprotective mechanisms rather than systemic inflammation itself. Although eHsp70 concentrations increased during the early stages of the experiment, its limited association with the remaining biomarkers indicates that its role in the regulation of iron homeostasis remains uncertain and warrants further investigation.

The inflammatory biomarkers formed a highly interconnected network of positive correlations. Particularly strong associations were observed between IL-1 and TNF-*α*, IL-1 and IL-6, and IFN-*γ* and IL-6. This pattern confirms that the analyzed biomarkers do not represent independent biological processes but rather different components of a coordinated inflammatory network. TNF-α and IL-1 are well-established early mediators of inflammation that induce the expression of numerous downstream inflammatory genes, including IL-6. Subsequently, IL-6 mediates many of the systemic effects of inflammation through its actions on the liver and the induction of acute-phase protein synthesis (8–10). The strong interrelationships among cytokines observed in the present study therefore provide additional evidence for the existence of a coordinated inflammatory response during the transition period. Similar correlation patterns have been reported in various experimental models of the acute-phase response (44, 45).

### 4.3 Modeling the effect of inflammatory parameters on parameters of iron metabolism

Unlike most previous studies, which have focused on individual inflammatory biomarkers and their separate relationships with metabolic parameters (15, 16, 24), the present study considered inflammation as an integrated biological process. The principal novelty of the present study is therefore not only the confirmation of the association between inflammatory activation and iron bioavailability, but also the demonstration that latent variable modeling can summarize coordinated inflammatory responses into a single biologically interpretable index. Compared with conventional analyses based on individual biomarkers, this approach better reflects the network nature of inflammation while minimizing the statistical problems arising from multicollinearity. An important aspect of the present findings is that the superior performance of PC1-infla should be interpreted in a statistical rather than causal context. The objective of developing the latent inflammatory index was not to imply that PC1 itself represents a biological determinant of iron metabolism, but rather to demonstrate that integrating multiple correlated inflammatory biomarkers into a single latent variable provides a more robust representation of the overall inflammatory response than considering each biomarker individually.

This approach is methodologically justified because inflammatory biomarkers do not represent independent processes; rather, they are components of a coordinated regulatory network that is activated simultaneously and collectively forms an inflammatory response (46, 47). In present study, correlation analysis revealed strong associations among nearly all inflammatory parameters, while the variance inflation factor (VIF) values for IL-1, TNF-*α*, and SAA exceeded the threshold indicative of substantial multicollinearity. Strong cascading interactions and synergistic effects among inflammatory mediators have also been reported in previous studies (48, 49). Under such circumstances, the application of conventional multivariate regression models may result in unstable coefficient estimates, inflated standard errors, and apparent changes in the direction of effects that do not accurately reflect the underlying biological processes (25).

PCA enabled the integration of correlated inflammatory signals into a single latent component representing the dominant pattern of variation. The first principal component, designated PC1-infla, explained 63.5% of the total variance, which is considered a high percent for complex biological systems (50). This finding indicates that most of the information contained in the individual inflammatory parameters can be represented by a single underlying inflammatory factor. The greatest contributions to this component were made by IL-1, TNF-*α*, and SAA, which is consistent with the established biology of acute inflammation. IL-1 and TNF-α are key initiators of the inflammatory response, whereas SAA is among the most sensitive systemic indicators of their activity (32). In contrast, the relatively small contribution of eHsp70 suggests that this biomarker reflects a distinct aspect of the cellular stress response and is not a central component of the inflammatory network regulating iron bioavailability.

The biological relevance of PC1-infla was confirmed at several levels. First, its temporal dynamics were characterized by an increase until the third day postpartum, followed by a gradual decline. This pattern closely mirrored the dynamics of the individual inflammatory parameters measured in the study and is consistent with the established course of postpartum inflammation in dairy cows (3–5). Consequently, PC1-infla can be interpreted as a biologically meaningful indicator of the overall inflammatory burden.

Second, a statistically significant effect of PC1-infla on iron-related parameters was demonstrated in the linear mixed-effects models. After accounting for temporal changes and individual differences among animals, PC1-infla was associated with all analyzed indicators of iron metabolism. These findings indicate that inflammation is not merely a parallel process accompanying the progression of early lactation, but rather an was independently associated with iron bioavailability. Previous experimental studies involving the administration of pro-inflammatory cytokines further support the central role of inflammation in the metabolic and physiological adaptation of dairy cows (51, 52).

An increase in PC1-infla was consistently associated with decreases in Fe, TIBC, UIBC, and TS%, suggesting the existence of a common regulatory mechanism affecting multiple aspects of iron metabolism simultaneously. Importantly, these effects were observed in all animals despite differences in baseline biomarker concentrations and response intensity, providing strong evidence that PC1-infla captures a fundamental physiological process characteristic of early lactation. The consistency of these associations across individual animals was further confirmed by spaghetti plots, which illustrated similar response patterns despite inter-animal variability. Such plots are widely recognized as valuable tools for evaluating longitudinal variation at the individual level in experimental studies (53, 54). These findings demonstrate that PC1-infla represents a robust measure of the overall inflammatory state of the organism. Rather than relying on a single cytokine or acute-phase protein, the latent inflammatory score captures the shared biological signal across multiple inflammatory biomarkers, reduces the influence of biomarker-specific variability, and effectively addresses the problem of multicollinearity. The importance of this approach is evident when comparing the results of univariate and multivariate analyses. In univariate models, individual inflammatory parameters often exhibit strong associations with iron metabolism because, in addition to their own biological effects, they partially capture the common inflammatory signal shared by other correlated biomarkers. In contrast, when multiple highly correlated inflammatory parameters are included simultaneously in multivariate models, the shared variance is statistically distributed among them. As a result, regression coefficients may become unstable and statistical significance may be inconsistently assigned to different biomarkers or lost altogether. Therefore, the PCA-derived latent inflammatory score provides a more biologically meaningful and statistically robust representation of the inflammatory process than analyses based solely on individual inflammatory markers (Figure 7). The scientific novelty of this research is not only reflected in the confirmation of the connection between inflammation and iron metabolism, but also in the demonstration that the latent PCA-based inflammatory index can be used as a robust biological and statistical tool to quantify the total inflammatory burden during early lactation.

**Figure 7:**
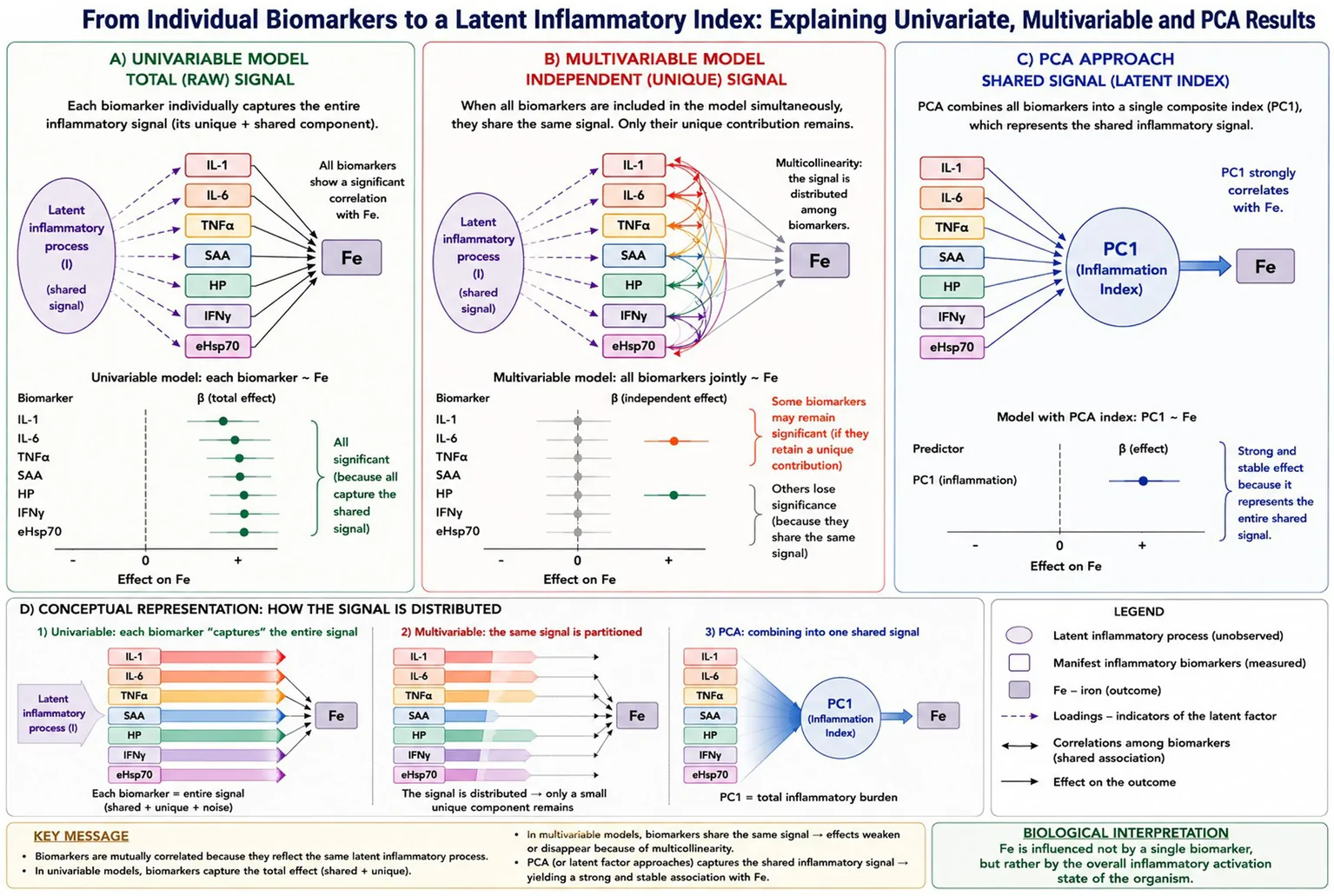
Conceptual diagram showing that a *PC1*-derived latent inflammatory index (*PC1*-infla) combines information from multiple correlated inflammatory biomarkers into a single measure representing the organism’s overall inflammatory state. The figure illustrates that the latent score captures the shared biological signal, reduces biomarker-specific variability and multicollinearity, and provides a more stable and biologically meaningful predictor than analyses using individual inflammatory markers, especially when comparing univariate and multivariate regression models.

Because this study was observational, the present findings should be interpreted as evidence of association rather than direct causality. Although the observed relationships are biologically consistent with current knowledge regarding inflammatory regulation of iron homeostasis, experimental studies including direct manipulation of inflammatory pathways will be necessary to establish causal mechanisms.

Instead of interpreting several correlated inflammatory biomarkers individually, a latent inflammatory index may provide a more comprehensive assessment of the overall inflammatory status during the transition period. The PCA-derived index may simplify statistical modeling by reducing redundancy among highly correlated inflammatory biomarkers. The proposed index may facilitate monitoring of the coordinated inflammatory response accompanying physiological postpartum adaptation. Following external validation, the proposed index may represent a useful research and monitoring tool for identifying cows experiencing excessive inflammatory responses during early lactation. The present findings support the concept that inflammatory regulation of iron homeostasis is better understood as a coordinated biological response rather than the effect of individual inflammatory mediators considered separately.

### 4.4 Study limitations

Several limitations of the present study should be acknowledged. First, the relatively small sample size limits statistical power and may reduce the generalizability of the findings. However, because each animal was sampled repeatedly throughout the postpartum period, the linear mixed-effects analyses were based on repeated observations rather than on a single measurement per cow. Such models are specifically designed for longitudinal data and provide efficient parameter estimation while accounting for the correlation among repeated measurements within animals. Therefore, the effective amount of information is greater than would be expected from the number of enrolled cows alone. Nevertheless, validation in larger populations remains necessary to confirm the robustness and generalizability of the proposed inflammatory index.

Second, all cows originated from a single commercial herd and were managed under standardized nutritional and environmental conditions. This design minimized variability arising from differences in herd management, feeding practices, housing, and environmental factors, thereby facilitating the investigation of biological relationships under controlled field conditions. At the same time, because the study was conducted in a single herd, the external validity of the findings should be interpreted with caution until the proposed inflammatory index is evaluated in independent herds with different management systems, breeds, and production conditions. As this represents the first study to investigate the relationship between a PCA-derived latent inflammatory index and iron bioavailability during early lactation, future multicenter validation studies will be an important next step.

Third, because the study was observational, the identified relationships should be interpreted as associations rather than evidence of direct causality. Nevertheless, the longitudinal repeated-measures design allowed us to characterize the temporal dynamics of inflammatory and iron-related biomarkers under physiological field conditions, providing a biologically relevant framework for generating mechanistic hypotheses. Experimental studies involving targeted modulation of inflammatory pathways will be required to establish causal relationships. Finally, hepcidin concentrations were not measured. Consequently, the proposed mechanistic interpretation relies on well-established physiological pathways together with the observed temporal associations, rather than on direct experimental confirmation of the IL-6–hepcidin–ferroportin axis. Future studies incorporating hepcidin measurements and experimental approaches will be essential to further validate the proposed mechanism.

Future studies involving larger populations, multiple herds, different breeds, and direct hepcidin determination are warranted.

## 5 Conclusion

The results of this study demonstrate that early lactation in dairy cows is characterized by coordinated activation of the inflammatory response accompanied by a simultaneous reduction in iron bioavailability. The PCA-derived latent inflammatory score (PC1-infla) successfully captured the shared inflammatory signal across multiple biomarkers and was significantly associated with of all analyzed indicators of iron bioavailability in the linear mixed-effects models. These findings suggest that the overall inflammatory burden may play a more important role in the regulation of iron metabolism than individual inflammatory markers alone. Consequently, latent inflammatory indices may represent valuable tools for investigating physiological and subclinical inflammation and its metabolic consequences in dairy cows.

The proposed index has potential significance as a research and monitoring tool for assessing postpartum inflammatory adaptation and identifying cows with excessive inflammatory responses during early lactation, pending further validation. Further investigations with larger animal groups, multiple herds and various breeds are needed to confirm its effectiveness and broader application.

## Data Availability

The raw data supporting the conclusions of this article will be made available by the authors, without undue reservation.
