# Supplementary material for: Latent inflammatory activity is associated with alterations in iron bioavailability during early postpartum adaptation in dairy cows
Source: Front Vet Sci. 2026 Sep 15;13:1923985. doi: 10.3389/fvets.2026.1923985 (PMC13619900; doi:10.3389/fvets.2026.1923985)

Suplementary Figure 1

Figure S1. Multiocolinearity (VIF value) between inflammatory parameters in experiment

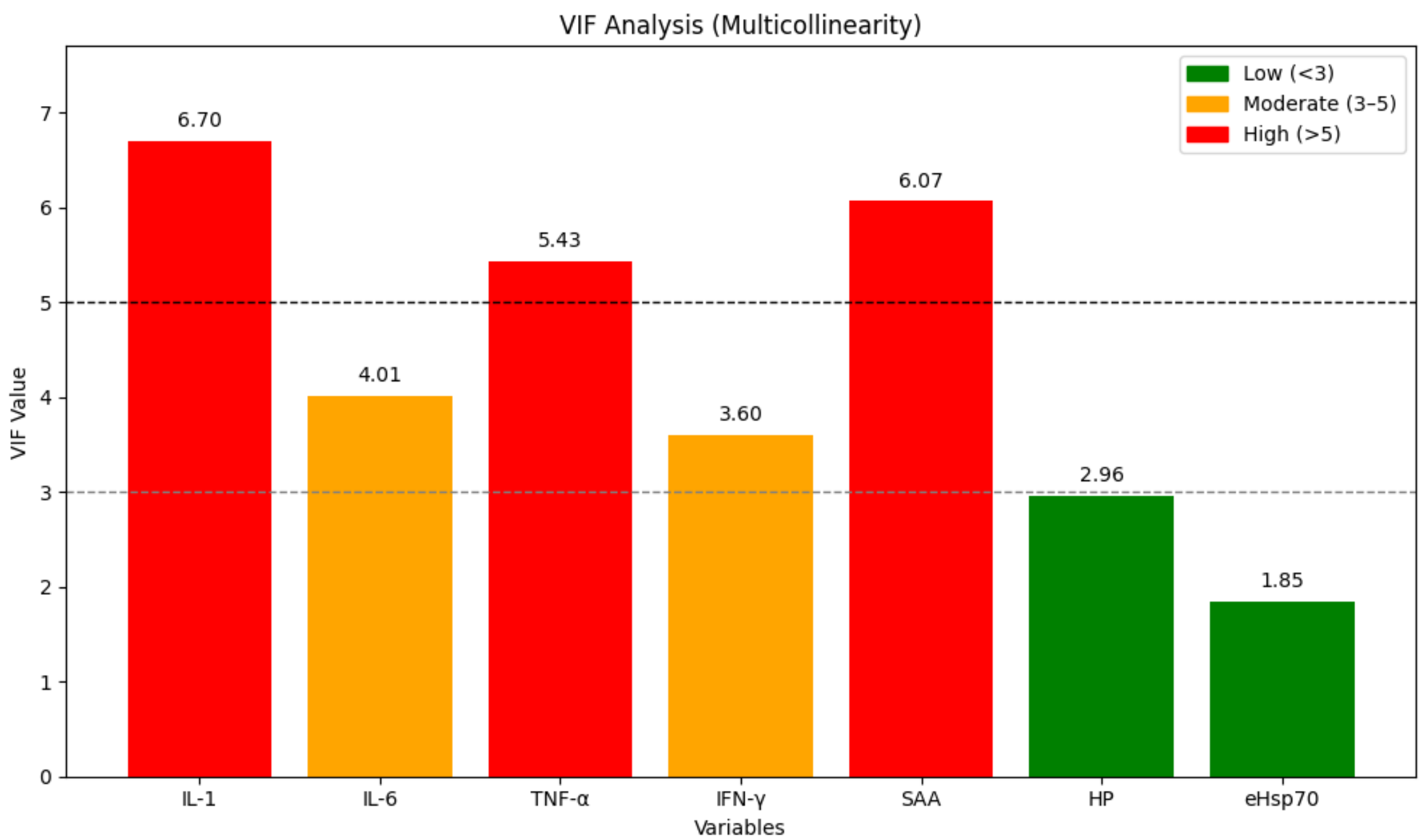

Supplement: Supplementary file 1 [file Image_1.pdf]
