# Supplementary material for: Latent inflammatory activity is associated with alterations in iron bioavailability during early postpartum adaptation in dairy cows
Source: Front Vet Sci. 2026 Sep 15;13:1923985. doi: 10.3389/fvets.2026.1923985 (PMC13619900; doi:10.3389/fvets.2026.1923985)
Supplement: Supplementary file 2 [file Table_1.DOCX]

Supplementary Table 1

Table S1. Influence of inflammatory parameters and inflammatory composite index on Fe, TIBC, UIBC and TS%

| **Fe** | **Model** | **Estimate** | **CI Lower** | **CI Upper** | **Std. Error** | **Statistic** | **P-value** |
| --- | --- | --- | --- | --- | --- | --- | --- |
| Univariate effect of inflammatory parameters on Fe value | (Intercept)  IL-1 | 28.2  -0.27 | 24.06  -0.35 | 32.34  -0.2 | 2.11  0.04 | 13.35  -6.99 | <0.001  <0.001 |
|  | (Intercept)  IL-6 | 26.96  -0.09 | 25.17  -0.11 | 28.75  -0.08 | 0.91  0.007 | 29.53  -9.77 | <0.001  <0.001 |
|  | (Intercept)  TNF-α | 23.34  -0.04 | 21.08  -0.06 | 25.6  -0.03 | 1.15  0.008 | 20.28  -4.46 | <0.001  <0.001 |
|  | (Intercept)  IFN-γ | 24.98  -0.04 | 22.86  -0.05 | 27.1  -0.03 | 1.08  0.005 | 23.05  -6.38 | <0.001  <0.001 |
|  | (Intercept)  SAA | 19.48  -0.02 | 18.37  -0.03 | 20.59  0 | 0.57  0.01 | 34.33  -2.42 | <0.001  0.018 |
|  | (Intercept)  HP | 24.4  -0.02 | 23.49  -0.02 | 25.32  -0.02 | 0.47  0.005 | 52.42  -18.26 | <0.001  <0.001 |
|  | (Intercept)  eHsp70 | 24.9  -2.19 | 21.47  -3.33 | 28.33  -1.05 | 1.75  0.58 | 14.23  -3.77 | <0.001  <0.001 |
| Multivariate effect of inflammatory parameters on Fe value | (Intercept)  IL-1  IL-6  TNF-α  IFN-γ  SAA  HP  eHsp70 | 26.59  -0.17  -0.04  0.03  0.03  0.01  -0.02  -0.09 | 23.1  -0.35  -0.07  -0.04  0.01  -0.02  -0.02  -0.87 | 30.08  0  -0.02  0.09  0.04  0.03  -0.01  0.69 | 1.78  0.09  0.01  0.03  0.01  0.01  0.004  0.4 | 14.94  -1.98  -3.25  0.77  4.62  0.49  -11.4  -0.23 | <0.001  0.051  0.002  0.443  <0.001  0.625  <0.001  0.816 |
| **TIBC** | **Model** | **Estimate** | **CI Lower** | **CI Upper** | **Std. Error** | **Statistic** | **P-value** |
| Univariate effect of inflammatory parameters on TIBC value | (Intercept)  IL-1 | 308.97  -0.65 | 297.88  -0.93 | 320.05  -0.36 | 5.65  0.15 | 54.64  -4.42 | <0.001  <0.001 |
|  | (Intercept)  IL-6 | 320.23  -0.38 | 311.56  -0.46 | 328.89  -0.3 | 4.42  0.04 | 72.43  -8.82 | <0.001  <0.001 |
|  | (Intercept)  TNF-α | 303.74  -0.16 | 293.16  -0.25 | 314.32  -0.08 | 5.4  0.05 | 56.26  -3.61 | <0.001  <0.001 |
|  | (Intercept)  IFN-γ | 315.86  -0.17 | 306.39  -0.21 | 325.32  -0.12 | 4.83  0.02 | 65.4  -6.91 | <0.001  <0.001 |
|  | (Intercept)  SAA | 289.55  -0.06 | 284.01  -0.11 | 295.1  0 | 2.83  0.03 | 102.31  -1.96 | <0.001  0.054 |
|  | (Intercept)  HP | 305.7  -0.06 | 299.68  -0.07 | 311.72  -0.05 | 3.07  0.01 | 99.48  -9.01 | <0.001  <0.001 |
|  | (Intercept)  eHsp70 | 305.85  -6.81 | 289.6  -12.13 | 322.1  -1.49 | 8.29  2.71 | 36.9  -2.51 | <0.001  0.014 |
| Multivariate effect of inflammatory parameters on TIBC value | (Intercept)  IL-1  IL-6  TNF-α  IFN-γ  SAA  HP  eHsp70 | 327.77  -1.59  -0.2  0.44  -0.01  0.03  -0.03  -1.81 | 303.41  -2.75  -0.36  0.01  -0.09  -0.13  -0.05  -7.22 | 352.13  -0.43  -0.04  0.88  0.07  0.19  -0.01  3.6 | 12.43  0.59  0.08  0.22  0.04  0.08  0.01  2.76 | 26.37  -2.68  -2.43  2  -0.26  0.37  -3.12  -0.66 | <0.001  0.009  0.018  0.049  0.795  0.714  0.003  0.513 |
| **UIBC** | **Model** | **Estimate** | **CI Lower** | **CI Upper** | **Std. Error** | **Statistic** | **P-value** |
| Univariate effect of inflammatory parameters on UIBC value | (Intercept)  IL-1 | 187.21  -0.74 | 179.83  -0.93 | 194.59  -0.54 | 3.76  0.1 | 49.74  -7.27 | <0.001  <0.001 |
|  | (Intercept)  IL-6 | 193.91  -0.36 | 188.67  -0.42 | 199.15  -0.31 | 2.68  0.03 | 72.48  -13.03 | <0.001  <0.001 |
|  | (Intercept)  TNF-α | 183.31  -0.21 | 176.23  -0.27 | 190.4  -0.14 | 3.61  0.03 | 50.74  -6.5 | <0.001  <0.001 |
|  | (Intercept)  IFN-γ | 190.21  -0.16 | 184.09  -0.19 | 196.34  -0.13 | 3.12  0.02 | 60.87  -9.86 | <0.001  <0.001 |
|  | (Intercept)  SAA | 166.81  -0.09 | 163.2  -0.13 | 170.41  -0.05 | 1.84  0.02 | 90.64  -4.22 | <0.001  <0.001 |
|  | (Intercept)  HP | 179.98  -0.06 | 176.66  -0.07 | 183.3  -0.05 | 1.69  0.003 | 106.34  -12.77 | <0.001  <0.001 |
|  | (Intercept)  eHsp70 | 187.45  -9.02 | 176.09  -12.79 | 198.8  -5.24 | 5.79  1.93 | 32.35  -4.68 | <0.001  <0.001 |
| Multivariate effect of inflammatory parameters on UIBC value | (Intercept)  IL1  IL6  TNFα  IFNγ  SAA  HP  eHsp70 | 201.42  -0.35  -0.18  0.05  0  0.01  -0.03  -2.52 | 188.63  -0.98  -0.28  -0.18  -0.04  -0.08  -0.04  -5.38 | 214.21  0.29  -0.09  0.29  0.04  0.1  -0.02  0.35 | 6.52  0.32  0.05  0.12  0.02  0.04  0.01  1.46 | 30.87  -1.07  -3.96  0.45  0.09  0.23  -5.78  -1.72 | <0.001  0.289  <0.001  0.654  0.928  0.821  <0.001  0.089 |
| **TS%** | **Model** | **Estimate** | **CI Lower** | **CI Upper** | **Std. Error** | **Statistic** | **P-value** |
| Univariate effect of inflammatory parameters on TS% value | (Intercept)  IL-1 | 44.9  -0.25 | 40.92  -0.36 | 48.88  -0.15 | 2.03  0.05 | 22.1  -4.62 | <0.001  <0.001 |
|  | (Intercept)  IL-6 | 48.64  -0.14 | 45.46  -0.17 | 51.81  -0.11 | 1.62  0.02 | 30.02  -8.24 | <0.001  <0.001 |
|  | (Intercept)  TNF-α | 43.22  -0.07 | 39.45  -0.1 | 46.99  -0.03 | 1.92  0.02 | 22.47  -4 | <0.001  <0.001 |
|  | (Intercept)  IFN-γ | 45.38  -0.05 | 41.75  -0.07 | 49.01  -0.03 | 1.85  0.01 | 24.51  -5.5 | <0.001  <0.001 |
|  | (Intercept)  SAA | 37.36  -0.02 | 35.52  -0.04 | 39.2  0 | 0.94  0.01 | 39.75  -2.06 | <0.001  0.043 |
|  | (Intercept)  HP | 45.05  -0.03 | 43.35  -0.03 | 46.75  -0.02 | 0.87  0.002 | 51.9  -15.33 | <0.001  <0.001 |
|  | (Intercept)  eHsp70 | 45.86  -3.39 | 40.18  -5.28 | 51.54  -1.5 | 2.9  0.96 | 15.82  -3.51 | <0.001  <0.001 |
| Multivariate effect of inflammatory parameters on TS% value | (Intercept)  IL-1  IL-6  TNF-α  IFN-γ  SAA  HP  eHsp70 | 47.73  -0.11  -0.06  -0.03  0.05  0.02  -0.03  -0.06 | 40.87  -0.45  -0.11  -0.15  0.03  -0.03  -0.04  -1.59 | 54.58  0.23  -0.01  0.1  0.07  0.06  -0.02  1.48 | 3.5  0.17  0.02  0.06  0.01  0.02  0.005  0.78 | 13.64  -0.64  -2.43  -0.43  4.6  0.74  -9.75  -0.07 | <0.001  0.523  0.017  0.67  <0.001  0.462  <0.001  0.942 |
